# Home Hospitalization for Acute Decompensated Heart Failure: Opportunities and Strategies for Improved Health Outcomes

**DOI:** 10.3390/healthcare6020031

**Published:** 2018-03-28

**Authors:** Konstantinos V. Voudris, Marc A. Silver

**Affiliations:** 1 Department of Medicine, University of Illinois at Chicago/Advocate Christ Medical Center, Oak Lawn, 4440 W 95th Street, Suite 131 NOB, Chicago, IL 60453, USA; dropsy1@me.com; 2 Division of Medical Services, Department of Medicine, Advocate Christ Medical Center, Oak Lawn, Chicago, IL 60453, USA

**Keywords:** home hospitalization, Acute Decompensated Heart Failure, hospital at home, health outcomes

## Abstract

Importance: Heart failure (HF) is the leading cause of hospitalization among patients over the age of 65 in the United States and developed countries, posing a significant economic burden to the health care systems. More than half of the patients with HF will be readmitted to the hospital within 6 months from discharge, leading not only to increased health care related expenses but also functional decline, iatrogenic injuries and in-hospital infections. With the increasing prevalence of HF, there is a substantial need for innovative delivery care models that can provide hospital level of care at a patient’s home. Observations: Home hospitalization was originally used to safely manage chronically ill patients with general medical (stroke, chronic obstructive pulmonary disease, deep vein thrombosis, community acquired pneumonia) and surgical conditions and was associated with improved patient satisfaction and improvement in activity of daily living status. This had no clear effect on readmission or cost. When hospital at home care model was applied to HF patients it demonstrated increased time to readmission, reduced index costs and improved health related quality of life, with no significant differences in adverse events. Eligible patients should be selected based on multiple factors taking into consideration applicable limitations and comorbidities. Conclusions and Relevance: Providing in-hospital level care to the patient’s house presents a reliable alternative, yielding multiple benefits both for the patient, as well as the health care system. Formulating a well-defined model is necessary before wide implementation.

## 1. Introduction

Heart failure (HF) is a life-threatening progressive disease, the leading cause of hospitalization among patients over the age of 65 in the United States and developed countries and remains an epidemic worldwide [1,2]. Patients with chronic HF typically have a course marked by worsening functional status, decreased mobility, progressively increased episodes of office, emergency department visits and acute care hospitalization; all are accompanied with a decline in health status and life quality.

Hospitalizations for HF represent a considerable burden to the health-care system and are responsible for more than 70% of the annual cost of HF care [3,4]. In a meta-analysis of 197 countries, covering 98.7% of the world’s population, the overall economic cost of HF in 2012 was estimated at $108 billion per annum. Direct costs accounted for ~60% ($65 billion) and indirect costs accounted for ~40% ($43 billion) of the overall spend [5]. Heart failure admissions account for 1–3% of all hospital admissions in the U.S. and the European countries, utilizing 1–2% of direct healthcare expenditure in Western industrialized countries [6]. In the USA, overall costs of heart failure in 2012 was estimated to be $30.7 billion. Of this total, 68% was attributable to direct medical costs [7]. By 2030, it is projected that prevalence of HF will increase by 46% compared to 2012, resulting in more than 8 million people, with HF. Subsequently, the total cost of care for HF is expected to increase by almost 127% to $69.7 billion from $30.7 billion in 2012 [8].

Despite the improvements in HF therapies, re-hospitalization rates remain high with more than 50% of the patients readmitted to the hospital within 6 months of discharge [9,10,11]. With the increasing prevalence of HF, there is a substantial need to explore innovative delivery care models, other than acute care hospitalization. These models will ideally provide optimal care and outcome, patient, family and provider satisfaction as well as reduced cost of health care provision and prevent disruption in heart failure care. As an important chronic disease, these strategies need to be similarly focused on longer term post-acute care outcomes and costs. [12]. Home-hospitalization provides significant opportunities to deliver on the needs of caring for HF patients more comprehensively and bears review and intense study.

## 2. Home Hospitalization–Definition

As the population continues to age, incidence and prevalence of chronic diseases in the general population increase, leading to elevated health care related expenses and further need for hospital beds. Multiple preventable adverse events have been previously reported to occur in the acute care hospital setting affecting primarily frail elderly patients, who commonly experience functional decline, iatrogenic injuries, delirium and in-hospital infections [13,14,15]. It may be possible to manage many of the patients initially admitted to the hospital, equally well with sufficient support outside the hospital environment, either to shorten hospital stays or to avoid hospital admittance [16]. In this context developing alternative systems to provide treatment for acute medical illness is critical.

Hospital at home (HaH) has been proposed as an alternative to hospitalization for patients with chronic illnesses that require a great deal of home care and have the highest readmission rate to acute care of all medical conditions. The rationale is that providing hospital ward-level or acute-level care in the patient’s home is a substitute for routine hospitalization, increasing patient and care giver satisfaction, improving quality of life and reducing the costs, without adverse effects on clinical outcomes.

Several care models have been described under the general definition of HaH, leading to conflicting results due to variances in patient populations, interventions and definitions. Leff and colleagues pursued a greater clarity of this definition beginning in 2004 [17]. HaH generally refers to the clinical activity of administering therapy and technology usually associated with acute inpatient care in a community setting. The spectrum of different types of care models may include outpatient intravenous infusion services, home-based nursing services and substitutive or ‘clinical unit model’ that delivers acute, hospital-level medical care in the home. Patients who have clinical indications for admission to a hospital ward are offered monitoring, face-to-face clinical care from nurses and physicians, diagnostic testing (e.g., laboratory investigations, electrocardiograms, radiography) and intravenous medication in their homes. This differs from most home-based models in its ability to handle high patient acuity and combine physician medical decision-making with a patient-tailored care team. Scalable substitutive models of hospital at home using virtual physician visit and remote biometric monitoring have been examined [18].

In order for a care model to meet the requirements for HaH model it needs to meet three substantial principles:

- It provides care that substitutes entirely for an inpatient acute hospital admission;
- It provides an intensity of care, including medical and nursing care, similar to that provided in the hospital;
- It provides care that cannot be provided by usual community-based home-care services [17].

HaH patients are those who, without the provision of the HaH service, would require inpatient care [19]. Rehabilitation programs, services providing long term care as well as palliative care should not be included under the HaH definition, as they are not designed to provide an intensity of care similar to that provided in the hospital.

Remote patient monitor (RPM) programs should not be considered as part of the HaH model as they are not designed to provide acute hospital level care. RPM programs use devices to remotely collect and send data to a health care facility for diagnostic interpretation or monitoring purpose. Data might be collected continuously or multiple times throughout the day and may include blood pressure, heart rate, electrocardiogram (ECG), or a variety of indicators for housebound patients. Such systems can be used to facilitate health care provided by physicians or home visiting nurses [20], although the effectiveness of RPM programs in reducing mortality or the number of cardiovascular hospitalizations compared with usual care is not currently established [21,22].

## 3. Home Hospitalization—Feasibility and Effectiveness

The effectiveness of HaH as a way of avoiding hospital admissions in patients with general medical and surgical conditions was first demonstrated by Shepperd et al. In their systematic review and meta-analysis of 10 randomized clinical trials in patients with medical conditions qualifying for inpatient hospitalization, they reported improved patient satisfaction, decreased mortality and reduced costs with HaH care compared to inpatient hospital care [23]. In a subsequent meta-analysis of 26 randomized controlled trials including patients with early discharge home for ongoing hospitalization, Shepperd et al. reported improved patient satisfaction but no clear effect on readmission rates or cost when compared to in-patient hospital care [24]. Both previously described meta-analyses included mainly patients with: stroke, pulmonary conditions (such as chronic obstructive pulmonary disease), deep vein thrombosis, community acquired pneumonia and other community acquired infections and less frequently patients with heart failure exacerbation. Finally, a third meta-analysis from Jeppesen et al. confirmed the efficacy of HaH in the management of acute exacerbations of chronic obstructive pulmonary disease. After analyzing eight trials with a total of 870 patients presenting with acute exacerbation of chronic obstructive pulmonary disease, they concluded that home treatment with support from respiratory nurses under guidance of the hospital medical team can be safely and successfully applied. Furthermore, they presented moderate quality evidence showing a trend towards reduced mortality and readmission rates, for patients with acute exacerbation of chronic obstructive pulmonary disease treated in a HaH setting, although these results did not reach statistical significance [25].

Other aspects of patient care have also been demonstrated to be influenced by the HaH model. Leff et al. investigated a subgroup of patients requiring acute hospital admission for an exacerbation of chronic obstructive pulmonary disease or HF, community-acquired pneumonia and cellulitis. At the end of their study they demonstrated HaH care to be associated with modestly better improvements in instrumental activities of daily living and trends toward more improvement in activity of daily living status than traditional acute hospital care. These findings may be facilitated by treatment in familiar environment and greater independence [26]. Additionally, on another study by Leff et al., HaH care was shown to be associated with lower levels of family member stress, when compared to traditional acute hospital care, without shifting the burden of care from hospital staff to family members [27]. Finally, adaptation of the HaH model can provide a possible solution for the acute care bed shortage most health systems face. In Australia, a construction equivalent to a 500-bed hospital following the HaH model, currently accommodates 33,000 admissions per year and accounts for almost 5% of all acute care beds in the state of Victoria, acting as a role model for other health systems to follow [28].

## 4. Home Hospitalization and Heart Failure

Despite the therapeutic advances, heart failure is associated with high morbidity and mortality, frequent hospital re-admission and increased costs related to hospitalization. In an attempt to improve medical care while reducing costs, both the European as well as the American HF guidelines recommend the implementation of management programs for patients with HF through an organized system of specialist HF care to improve symptoms, reduce hospitalizations and increase survival [1,29]. Outpatient follow-up within 7 days after discharge from a heart failure hospitalization has been widely implemented after recognition that nearly half of HF-readmissions occurred before the first ambulatory visit [30]. In general, these programs are initiated after hospitalization and usually incorporate enhanced patient self-care, follow-up monitoring by specially trained staff and access to specialized HF clinics [31]. The two most widely accepted programs are HF clinics and home-based care programs, which start during admission or just after discharge [32]. In both programs patients typically receive nurse-led multidisciplinary care with minimal to no physician participation.

With the reported effectiveness of HaH model in patients with general medical and surgical conditions and the undoubtable benefits of multidisciplinary outpatient management strategies in HF patients, the question of extending HaH in HF patients arose. Providing inpatient level of care in the patient’s home, in a frail population with frequent hospitalizations appeared to have multiple benefits both for the patient, as well as the health care system. Patel et al. were the first to evaluate the feasibility and effectiveness of a physician-led hospital-at home service for selected elderly patients with acute decompensation of chronic heart failure. They randomized 31 patients with decompensated HF to home care or conventional treatment. Patients assigned to home care were discharged from the hospital within 48 h and were followed up in their homes by specialist nurses, while having telephonic access to cardiology physicians. At 1-year follow up of this small group of patients, no significant differences were identified in adverse events or in health-related quality of life between the two procedures but there was a significant reduction in cost with the use of home care [33]. Likewise, Tibaldi et al. randomly assigned 75 elderly patients with acute de-compensation of HF to geriatric HaH service (*n* = 48) or to the general medical ward (*n* = 53). At 6 moths follow up, mortality and number of subsequent hospitalizations were not significantly different between the two groups, while mean time to first additional admission was significantly longer for the geriatric HaH service group. Interestingly, geriatric HaH service patients experienced improvements in depression, nutritional status and quality-of-life scores [34]. The notion of HaH in HF patients was further supported by Mendoza et al. In their study, they randomly assigned 80 patients presenting to the Emergency Department with decompensated HF to either HaH care, consisting of physicians and nurses, undertaking visits to the patient’s home, or hospital care. At 1 year follow up, HaH care allowed an important reduction in costs during the index episode compared to hospital care, while maintaining similar outcomes with respect to cardiovascular mortality and morbidity and quality of life [35].

These findings were confirmed by a recent meta-analysis by Qaddoura et al. using 3 randomized controlled studies (*n* = 203) and narratively synthesized results from 3 observational studies (*n* = 329) [36]. In randomized controlled studies, HaH increased time to first readmission (mean difference (MD) 14.13 days (95% CI 10.36 to 17.91)) and improved health-related quality of life at both, 6 months (standardized mean difference (SMD) −0.31 (−0.45 to −0.18)) and 12 months (SMD −0.17 (−0.31 to −0.02)). Furthermore, in randomized controlled studies, HaH demonstrated a trend to decreased readmissions (risk ratio (RR) 0.68 (0.42 to 1.09)) and had no effect on all-cause mortality (RR 0.94 (0.67 to 1.32)). Additionally, HaH decreased costs of index hospitalization in all randomized controlled studies. Finally, HaH reduced readmissions and emergency department visits per patient in all 3 observational studies included in the meta-analysis (Table 1).

## 5. Patients’ Selection

Selecting the subset of HF population expected to benefit the most from HaH model is a challenge for this innovative approach. HF patient population includes patients with different ent aetiologies of reduced heart function, variable functional capacity and associated comorbidities, requiring an individualized approach in their care. Setting robust eligibility criteria for triage into HaH care is substantial to avoid triggering utilization of these services from patients who may not benefit from them. It is important to emphasize that most studies evaluating home-based chronic HF programs have used strict inclusion criteria incorporating only a small proportion of the initially screened population.

Various factors have been shown to affect the risk profile for individuals with acute decompensated HF. Older age, lower systolic blood pressure, higher respiratory rate, higher blood urea nitrogen level and hyponatremia were all found to be predictors of 30-day mortality after admission for acute decompensated HF in the Enhanced Feedback for Effective Cardiac Treatment (EFFECT) study [37]. Subsequently published reports from the Outcomes of a Prospective Trial of Intravenous Milrinone for Exacerbations of Chronic Heart Failure (OPTIME-CHF) [38] and the Acute Decompensated Heart Failure National Registry (ADHERE) [39] reaffirmed the prognostic importance of systolic blood pressure, blood urea nitrogen and/or serum creatinine concentration and hyponatremia for death occurring within 60 days from presentation. Other adverse prognostic factors associated with 6-month mortality reported in the Evaluation Study of Congestive Heart Failure and Pulmonary Artery Catheterization Effectiveness (ESCAPE) study included clinical instability defined by cardiac arrest, the need for mechanical ventilation, intolerance to beta-blocker therapy and significant functional limitations, defined by a short 6-minute walk distance [40].

In the available studies [33,34,35,36], HaH model was applied in patients with confirmed HF presenting to the emergency department with worsening dyspnea and/or worsened pulmonary or systemic congestion symptoms manifesting decompensation of chronic HF. New York Heart Association (NYHA) class ranged between II and IV depending on the study. Patients were offered the option to receive care at home only after undergoing the appropriate cardiac work up in the Emergency department and evaluation by a heart specialist. Appropriate care supervision at home and telephone connection were also required prior to discharge at home. In order to ensure the patients’ safety and minimize the risks of relapse, patients with new-onset heart failure, severe co-morbidities (e.g., malignancy, liver failure) or other factors such as acute psychiatric disease, confusion, alcohol abuse and hemodynamic instability were not eligible for treatment in home hospital. Exclusion criteria further precluded patients with electrolyte disturbances that might cause arrhythmias, severe renal failure and acute ischemic events. Finally, some patients preferred being visited at home, whereas others preferred the idea of leaving their house, talking to other patients and seeing a range of health care workers at their local hospital [41].

Risk stratification models during HF evaluation can assist with appropriate allocation of resources. Identification of low risk patients could allow for discharge directly from the Emergency department or Observation Unit whereas higher risk patients with multiple risk factors associated with adverse outcomes would necessitate inpatient or HaH care and close outpatient follow up. Prognostic models based on the available data from hospitalized patients in the ESCAPE, ADHERE and EFFECT clinical studies have been developed. The ESCAPE model utilizes age, BUN, 6 min walk <300 feet, sodium level <130, CPR/mechanical ventilation, diuretic dose and b-blocker use at discharge and discharge BNP level [40]. Similarly, the EFFECT model utilizes age, systolic blood pressure, respiratory rate, sodium level, BUN level and comorbid conditions (cerebrovascular disease, dementia, COPD, hepatic cirrhosis, cancer) [37], whereas the ADHERE model utilizes BUN level, systolic blood pressure, age, heart rate and serum Creatinine level [39]. All three models demonstrated equally predictive ability in identifying patient cohorts with high, medium or low risk for death in 30 days or 6 months. Finally, the Emergency Heart Failure Mortality Risk Grade (EHMRG) is unique compared to other validated risk prediction models as it was derived in a broad cohort of patients with acute decompensated HF presenting to the ED who were either discharged home or admitted to the hospital [42]. The score combines age, systolic blood pressure, heart rate, oxygen saturation, serum creatinine, serum potassium, serum troponin, presence of active cancer, current use of metolazone and mode of arrival to the ED to estimate 7-day mortality after presenting to the ED with acute decompensated HF. Those in the lowest two risk quantiles demonstrated exceedingly low 7-day mortality rate suggesting a possible role in the initial evaluation and placement of patients with acute decompensated HF.

Never the less, none of the described models are currently suitable for wide spread application in the evaluation and triage of HF patients. Subsequently their use in patient selection for HaH is currently limited by lack of available studies regarding their effectiveness and prognostic ability in that setting. Further prospective validation of the models in independent databases, daily clinical practice and in clinical trials is required.

## 6. Limitations

The current data on HaH model implementation in HF patients have several limitations. All studies reported included a small number of patients with very specific characteristics. Thus, conclusions cannot be extrapolated to all HF patients. Despite randomization, in many studies, there were statistically significant differences in some variables between the in-study groups, potentially affecting the reported outcomes. However, reliable adjustment was impossible due to small sample size. Additionally, most of the studies explored the impact of the intervention and did not analyze the incremental benefits of the various components of each intervention due to lack of direct comparisons. Moreover, all studies evaluating HaH model in HF patients have been relatively short in duration, raising questions about their long-term effectiveness. Finally, prospective validation of risk stratification models in the HaH setting is required prior to widespread utilization.

## 7. Looking Forward

The review above focuses on the signals to date on the viability of HaH as a therapeutic substitution strategy for chronic heart failure decompensation events. However, the current juxtaposition of the explosion of heart failure, in specific and all chronic disease patient populations, along with a healthcare crisis in terms of cost and quality of care appear to be driving more intensified interest in alternative models of care for the acutely decompensated patient. We envision the home as having the potential beyond a site of care for acute decompensation; with the ability to flex into a site for rehabilitation, nutrition, psychosocial support, continued medicine up-titration and ongoing education towards complete health care literary. In this motion, the home may become the driver for delivery of cost efficient yet evidence based affordable and acceptable health care.

**Figure 1:**
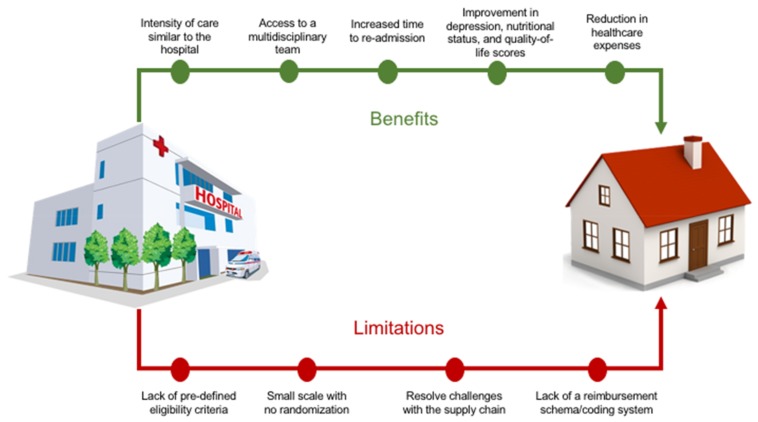
Central Illustration: Benefits and limitations of home hospitalization model.

Additionally, multiple key infrastructure pieces, heretofore sub optimally operationalized or not yet envisioned, need to be hard wired to help create this important step in health care delivery. Included are:

- Resolve challenges with the supply chain going into the home (it is currently tuned for a “post-acute” versus “acute care” reality).
- Lack of a readily accessible reimbursement schema/coding system that aligns the clinical, financial and satisfaction benefits of the model with the current payment system. Current health care financing does envision a “bundled” care plan but not a comprehensive, single episode of care based in a patient’s home.
- The critical need to integrate the restorative phase of care with the acute phase using a single care team. A common failure mode is the step off in coordination and understanding of the patients (and family’s) needs to truly recover and prevent future acute exacerbations. There are currently significant limitations in care management and transition plans difficult to overcome in current models (Figure 1).

Finally, a shift in healthcare reimbursement approaches is needed to align with the other incentives offered for home hospitalization [43].

## 8. Conclusions

The design and goals of chronic HF management vary according to multiple variables, including the provider setting, patient capacity for self-management and the severity of disease. Recurrent hospitalizations remain a marker for disease severity, quality of life and prognosis. Providing in-hospital level care to the patient’s house presents as a reliable alternative, yielding multiple benefits both for the patient as well as the health care systems. The most important components for the success of home care programs are (1) identifying the population that can benefit the most from this intervention (and those who will not) (2) providing ready access to a multidisciplinary team consisting of both specialists and nurses (3) formulating a well-defined model offering services only to those who need it and (4) establishing and innovating around healthcare economics with billing codes that would facilitate and promote novel care delivery. Further development of HaH care will require additional research with new large multicenter, randomized controlled trials and also close attention to incubator models that emit strong signals of learning.

## Figures and Tables

**Table 1:** Synopsis of main studies investigating home-based care programs for heart failure.

Author	Study	Study Population	Finding
Patel H. et al. [33]	Home care as an option in worsening chronic heart failure—a pilot study to evaluate feasibility, quality adjusted life years and cost-effectiveness.	31 patients seeking medical attention at hospital for worsening CHF were randomized to home care or conventional care.	Reduction in cost of care for selected patients with CHF eligible for hospital care might be achieved by early discharge from hospital followed by home visits.
Tibaldi V. et al. [34]	Hospital at home for elderly patients with acute decompensation of chronic heart failure: a prospective randomized controlled trial.	101 patients randomly assigned to the general medical ward (*n* = 53) or to the Geriatric Home Hospitalization Service (*n* = 48).	(a) Substitutive hospital-at-home care is a viable alternative to traditional hospital inpatient care for elderly patients with acutely decompensated CHF. (b) No difference in mortality at 6 months and number of subsequent hospital admissions was observed. (c) Geriatric Home Hospitalization Service patients experienced improvements in depression, nutritional status, quality-of-life scores and had a longer time to first additional admission.
Mendoza H. et al. [35]	'Hospital at home’ care model as an effective alternative in the management of decompensated chronic heart failure.	Eighty patients over the age of 65 years who presented at the emergency department with decompensated HF randomly assigned to inpatient hospital care or Hospital at Home.	Hospital at home care allows an important reduction in the costs during the index episode compared with hospital care, whilst maintaining similar outcomes with respect to cardiovascular mortality and morbidity and quality of life at 1 year follow-up.
**Meta-Analysis**			
Qaddoura A. et al. [36]	Efficacy of Hospital at Home in Patients with Heart Failure: A Systematic Review and Meta-Analysis.	Meta-analyzed results from 3 RCTs (*n* = 203) and narratively synthesized results from 3 observational studies (*n* = 329).	Hospital at Home appears to increase time to readmission, reduce index costs and improve health-related quality of life among patients requiring hospital-level care for HF.
